# ANT1-mediated fatty acid-induced uncoupling as a target for improving myocellular insulin sensitivity

**DOI:** 10.1007/s00125-016-3885-8

**Published:** 2016-02-17

**Authors:** Lauren M. Sparks, Anne Gemmink, Esther Phielix, Madeleen Bosma, Gert Schaart, Esther Moonen-Kornips, Johanna A. Jörgensen, Emmani B. M. Nascimento, Matthijs K. C. Hesselink, Patrick Schrauwen, Joris Hoeks

**Affiliations:** Department of Human Biology, NUTRIM School of Nutrition and Translational Research in Metabolism, Maastricht University, P. O. Box 616, 6200MD Maastricht, the Netherlands; Translational Research Institute for Metabolism and Diabetes, Florida Hospital, Orlando, FL USA; Department of Human Movement Sciences, NUTRIM School of Nutrition and Translational Research in Metabolism, Maastricht University, Maastricht, the Netherlands; Department of Cell and Molecular Biology, Karolinska Institutet, Stockholm, Sweden

**Keywords:** ANT1, Fatty acid-induced uncoupling, Skeletal muscle insulin sensitivity

## Abstract

**Aims/hypothesis:**

Dissipating energy via mitochondrial uncoupling has been suggested to contribute to enhanced insulin sensitivity. We hypothesised that skeletal muscle mitochondria of endurance-trained athletes have increased sensitivity for fatty acid (FA)-induced uncoupling, which is driven by the mitochondrial protein adenine nucleotide translocase 1 (ANT1).

**Methods:**

Capacity for FA-induced uncoupling was measured in endurance-trained male athletes (T) and sedentary young men (UT) in an observational study and also in isolated skeletal muscle mitochondria from Zucker diabetic fatty (ZDF) rats and C2C12 myotubes following small interfering RNA (siRNA)-mediated gene silencing of ANT1. Thus, fuelled by glutamate/succinate (fibres) or pyruvate (mitochondria and myotubes) and in the presence of oligomycin to block ATP synthesis, increasing levels of oleate (fibres) or palmitate (mitochondria and myotubes) were automatically titrated while respiration was monitored. Insulin sensitivity was measured by hyperinsulinaemic–euglycaemic clamp in humans and via insulin-stimulated glucose uptake in myotubes.

**Results:**

Skeletal muscle from the T group displayed increased sensitivity to FA-induced uncoupling (*p* = 0.011) compared with muscle from the UT group, and this was associated with elevated insulin sensitivity (*p* = 0.034). ANT1 expression was increased in T (*p* = 0.013). Mitochondria from ZDF rats displayed decreased sensitivity for FA-induced uncoupling (*p* = 0.008). This difference disappeared in the presence of the adenine nucleotide translocator inhibitor carboxyatractyloside. Partial knockdown of ANT1 in C2C12 myotubes decreased sensitivity to the FA-induced uncoupling (*p* = 0.008) and insulin-stimulated glucose uptake (*p* = 0.025) compared with controls.

**Conclusions/interpretation:**

Increased sensitivity to FA-induced uncoupling is associated with enhanced insulin sensitivity and is affected by ANT1 activity in skeletal muscle. FA-induced mitochondrial uncoupling may help to preserve insulin sensitivity in the face of a high supply of FAs.

***Trial registration*:**

www.trialregister.nl NTR2002

## Introduction

Endurance exercise training has beneficial effects on mitochondrial oxidative capacity in skeletal muscle and is associated with enhanced muscle insulin sensitivity. We and others have previously shown that endurance and resistance exercise training programmes exert beneficial effects on muscle substrate metabolism in individuals with type 2 diabetes [1–3], and that a high skeletal muscle oxidative capacity (due to chronic endurance exercise training) partially protects against lipid-induced insulin resistance [4].

Interestingly, resting muscle of endurance-trained athletes is characterised by inefficient mitochondrial coupling (higher basal uncoupling rates). Using in vivo MR spectroscopy, a 54% higher basal rate of substrate oxidation through the tricarboxylic acid (TCA) cycle was observed in athletes compared with matched sedentary controls [5]. Despite this increased basal TCA cycle flux, ATP synthesis rates were similar, thus indicating a decrease in the efficiency of mitochondrial coupling. The mechanistic basis for the decreased mitochondrial coupling efficiency in endurance-trained individuals, however, has not been established. The study [5] concluded that increased mitochondrial uncoupling may represent an additional mechanism by which endurance exercise training enhances muscle insulin sensitivity; in accordance, muscle-specific overexpression of uncoupling protein (UCP) 1 [6] and UCP3 [7] in rodents protect against lipid-induced insulin resistance.

We have previously shown that UCP3 expression is lower in oxidative type I muscle fibres [8, 9] than in the more glycolytic type II fibres, and *UCP3* mRNA expression and protein abundance are, in fact, reduced in muscle from endurance-trained humans [9, 10]. Employing genetic and physiological mouse models for gain- and loss-of-function of skeletal muscle UCP3, we previously failed to find evidence for any involvement of UCP3 in mediating basal mitochondrial uncoupling [11, 12]. Alternatively, it has been known for decades that fatty acids (FAs) can affect mitochondrial efficiency due to their ability to induce mitochondrial uncoupling [13]. Although the mechanisms underlying FA-induced uncoupling are not yet fully understood, there are clear indications that the mitochondrial protein adenine nucleotide translocator 1 (ANT1)—in addition to its function in mitochondrial ADP–ATP exchange—can also transport FA anions across the inner mitochondrial membrane [13, 14] and thus mediate FA-induced uncoupling.

Skeletal muscle from endurance-trained individuals shows enlarged intramyocellular lipid stores, high FA fluxes and increased *ANT1* (also known as *SLC25A4*) mRNA and protein abundance compared with muscle from matched sedentary individuals [4, 10, 15, 16]. We tested the hypothesis that endurance-trained muscle is more sensitive to the uncoupling effect of FAs and that FA-induced uncoupling plays a significant role in insulin sensitivity. To further study the role of ANT1 in FA-induced uncoupling in relation to insulin responsiveness, we also examined isolated skeletal muscle mitochondria obtained from a rodent model of type 2 diabetes (Zucker diabetic fatty [ZDF] rats) and employed a small interfering RNA (siRNA)-mediated gene silencing of *Ant1* in C2C12 myotubes.

## Methods

### Ethical approval

The human studies were approved by The Medical Ethical Committee of Maastricht University and conformed to the Declaration of Helsinki. Participants gave informed written consent. Animal experiments were approved by the Institutional Animal Care and Use Committee of Maastricht University and complied with the principles of laboratory animal care.

### Human participants

Ten healthy, young, sedentary men (U) and nine endurance-trained ($\overset{.}{V}{\mathrm{O}}_{2\mathrm{peak}}$ >55 ml kg^−1^ min^−1^) male athletes (T) were included in this study as previously described [4]. Data on lipid-induced insulin sensitivity was published previously [4]. Briefly, each participant underwent two test days on which insulin sensitivity was measured during a 6 h hyperinsulinaemic–euglycaemic (40 mU m^−2^ min^−1^) clamp according to DeFronzo [17]. Two clamps were performed on different occasions with either co-infusion of glycerol (control) or an emulsion of long-chain triacylglycerols (Intralipid; Baxter, Utrecht, the Netherlands). A muscle biopsy was taken from the vastus lateralis [18] prior to infusion. Plasma glucose and insulin concentrations and isotopic enrichment of plasma glucose were determined as previously described [19].

### Animals

Male, leptin receptor-deficient ZDF rats (ZDF/Gmi, *fa*/*fa*, *n* = 4) and homozygous lean rats (ZDF/Gmi, *+*/*+*, *n* = 4) were purchased from Charles River Laboratories (Maastricht, the Netherlands). Animals were housed in pairs of one genotype on a 12–12 h light–dark cycle at room temperature (21–22°C) with free access to food (Purina 5008; Purina Mills, St. Louis, MO, USA) and water. At 14 weeks of age, rats were killed by cervical dislocation. Gastrocnemius muscle was rapidly dissected and placed into ice-cold mitochondrial isolation medium containing 100 mmol/l sucrose, 50 mmol/l KCl, 20 mmol/l K^+^-TES, 1 mmol/l EDTA and 0.2% (wt/vol.) FA-free BSA for isolation of mitochondria.

### Cell culture

C2C12 cells (Sol8, ATCC CRL-2174) were purchased from LGC Standards (Teddington, Middlesex, UK). LGC Standards provided authentication. The Sol8 cell line is a myogenic cell line isolated by Daubas et al [20] from primary cultures of soleus muscle taken from the leg of a normal C3H mouse. The C2C12 cells were then evaluated in-house at Maastricht University and tested negative for mycoplasma. The cells were maintained in DMEM with 10% FBS and differentiated for 7 days in DMEM with 2% FBS. At day 6 of differentiation, cells were transfected with 10 nmol/l Stealth RNAi oligos using Lipofectamine RNAiMAX (Invitrogen, Breda, the Netherlands) as transfection reagent for ANT1. Scrambled oligonucleotides were used as the negative control. Cells were harvested at 24 h post transfection.

### Muscle fibre preparation

Human muscle fibres (~20 mg) were separated using small needles in conservation medium (BIOPS; Oroboros Instruments, Innsbruck, Austria), and the muscle membrane was permeabilised with saponin as previously described [21]. Subsequent to washing with respiration medium (MiR05; Oroboros Instruments), ~3–4 mg wet-weight fibres were transferred into an Oxygraph (Oroboros Instruments) for oxygen consumption measurements.

### Mitochondria isolation

Rat left gastrocnemius muscle was rapidly excised and placed into ice-cold mitochondrial isolation medium and mitochondria isolated as previously described from only one animal per experimental day [11] on alternating days for ZDF and wild-type controls. The concentration of mitochondrial protein was measured using fluorescamine (Fluram; Fluka, Zwijndrecht, the Netherlands) with BSA as a standard [22]. Isolated mitochondria were used for oxygen consumption measurements. Remaining mitochondria were stored with protease inhibitor cocktail (Complete Mini; Roche, Sigma Aldrich/Fluka, the Netherlands) for additional protein analyses.

### Glucose uptake

On day 7 of differentiation, C2C12 myotubes (~1 × 10^6^) were trypsinised and centrifuged at 218 *g* for 5 min at room temperature. The supernatant fraction was removed and cells re-suspended in 2.3 ml of MiR05. Two millilitres of the cell suspension was added to the Oxygraph and the remaining 0.3 ml was used for protein determination for normalisation. RIPA buffer was added to the remaining cell suspension, sonicated and centrifuged at 21,672 *g* for 10 min at 4°C and the supernatant fraction was frozen at −80°C for western blot analyses. Basal and insulin-stimulated deoxyglucose uptake assays were performed as previously described [23] and in quadruplicate.

### Oxygen consumption measurement

All oxygen consumption measurements were performed in duplicate. Capacity for FA-induced uncoupling in permeabilised muscle fibres, mitochondria or permeabilised myotubes was measured in MiR05 in the presence of 10 mmol/l glutamate plus 10 mmol/l succinate (fibres) or 5 mmol/l pyruvate (mitochondria and myotubes) as substrates, and oligomycin (1 μg/ml) to block ATP synthesis, at 37°C. In isolated mitochondria and myotubes, increasing levels of palmitate were then automatically titrated by a pump (Oroboros Instruments) while respiration was monitored. In isolated mitochondria from ZDF rats, this measurement was performed in the absence and presence of 0.5 μmol/l of the ANT inhibitor carboxyatractyloside (CATR).

In human skeletal muscle fibres, higher FA concentrations were required to achieve maximal FA-induced uncoupling, which resulted in unstable respiration rates when using palmitate. Since this problem was absent when using oleate, FA-induced uncoupling in human permeabilised fibres was assessed using oleate. Previous work demonstrated that both FAs generally have similar qualitative effects on uncoupling [12].

Concentrations of unbound palmitate were calculated as previously described [24]. Data for FA concentration–response curves were analysed with the four parameter logistic curve fit option of the Sigmaplot 8.0 application (SigmaPlot, Erkrath, Germany). ADP-stimulated and oligomycin-insensitive respiration were assessed as previously described [25] with 5 mmol/l pyruvate or 50 μmol/l palmitoyl-CoA plus 2 mmol/l carnitine. Due to significant differences in mtDNA copy number between T and UT, we normalised all respiration data from muscle fibres to mtDNA copy number.

### RNA isolation and qRT-PCR

Total RNA was isolated from ~20 mg of human skeletal muscle tissue using the acid phenol method [26]. Total RNA was isolated from C2C12 myotubes as previously described [27]. RNA quantity was confirmed with the NanoDrop (NanoDrop Technologies, Wilmington, DE, USA). *ANT1*, Rn18s and *GAPDH* primers were obtained from Applied Biosystems (Roche, Branchburg, NJ, USA). Real-time quantitative RT-PCR (qRT-PCR) reactions were performed as one-step reactions on the ABI PRISM 7900 real-time PCR system from Applied Biosystems (Nieuwerkerk aan den Ijssel, the Netherlands) as previously described [28]. SYBR Green was used as the reporter dye. For all assays performed in human samples, *GAPDH* was the reference gene, and for all assays performed on C2C12 myotubes, *Rn18s* was the reference gene. All expression data were normalised by dividing the target gene by the reference gene. All qRT-PCR measurements were performed in triplicate.

### Western blotting

Western blot analyses were performed in whole lysates of human muscle tissue and C2C12 myotubes and in isolated skeletal muscle mitochondria (wild-type and ZDF rats). Coomassie Brilliant Blue staining was used to determine total protein content. Analyses were performed using 10% polyacrylamide-SDS gels as previously described [29, 30]. Equal protein loading was confirmed by western blotting of porin/voltage-dependent anion channel (VDAC) for isolated mitochondria and α-actin for tissue and cells. Monoclonal antibodies against ANT1 (MSA02; Mitosciences, Eugene, OR, USA; mouse IgG; dilution 1:2,000), α-actin for tissue (sc-58670; Santa Cruz, CA, USA; mouse IgM; dilution 1:10,000), β-actin for cells (A-5316; Sigma-Aldrich, St. Louis, MO, USA; mouse IgG1; dilution 1:25,000) and a polyclonal antibody against porin/VDAC (sc-8828; Santa Cruz, CA, USA; goat IgG; dilution 1:10,000) were used. For validation, we used a protein marker (Precision Plus All Blue Prestained Protein Standards from Bio-Rad Laboratories, Veenendaal, the Netherlands; no. 1610373) on the same blots. All of these commercially available antibodies showed a single distinct band at the molecular weight indicated in the datasheets. Bands of interest were detected and quantified with Odyssey Infrared Imager (LI-COR, Leusden, the Netherlands).

### Statistical analyses

Data are presented as mean ± SEM. Statistical analyses were performed using SPSS v16.0.2 for Mac (Chicago, IL, USA) and GraphPad Prism v5.0 (San Diego, CA, USA). Differences between groups were analysed by unpaired Student’s *t* tests. Pearson’s correlation coefficients were used to describe the linear association between variables. FA concentrations were log-transformed for traces where necessary and indicated in the figure legends appropriately. Statistical significance was set at *p* < 0.05.

## Results

### Insulin sensitivity and FA-induced uncoupling in T and UT

**Fig. 1:**
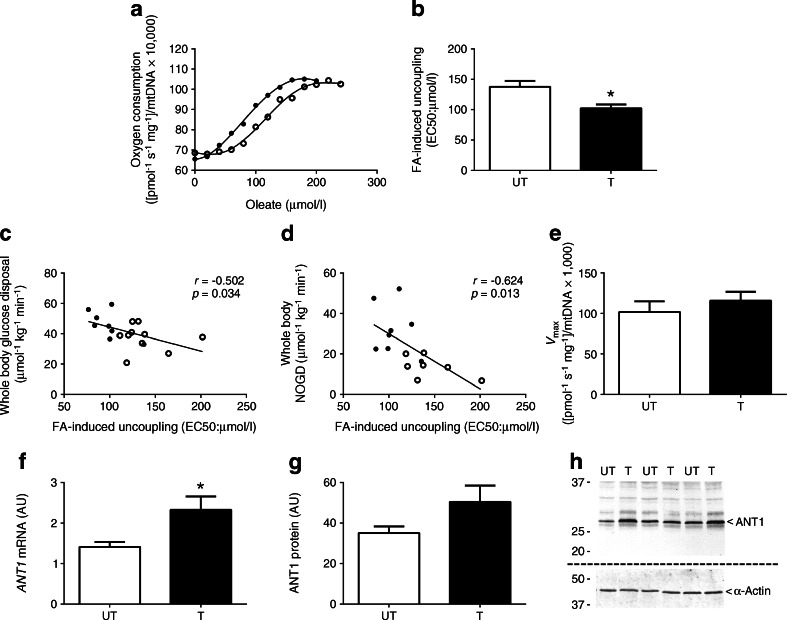
(**a**) Typical experiment assessing FA-induced (oleate) uncoupling in muscle fibres of healthy UT and T. (**b**) Sensitivity to FA-induced uncoupling (EC50) is shown in UT and T. (**c**, **d**) FA-induced uncoupling correlated with insulin sensitivity (whole-body glucose disposal) (**c**) and insulin-stimulated NOGD (**d**). (**e**) Maximal oleate-induced uncoupling (*V*_max_) in permeabilised muscle fibres from UT and T. (**f**, **g**) *ANT1* mRNA expression (**f**) and ANT1 protein abundance (**g**) in skeletal muscle tissue of UT and T (shown in arbitrary units [AU]). (**h**) Representative blots for protein abundance. Two separate gels and blots were used for ANT1 and α-actin as indicated by the dashed line. *GAPDH* was used as a reference gene for mRNA expression. α-Actin was used to quantify equal protein loading during the western blot analysis. White circles and bars, UT (*n* = 10); black circles and bars, T (*n* = 9). Data are presented as mean ± SEM. *p < 0.05, T vs UT

We determined FA-induced uncoupling in skeletal muscle fibres from nine T and ten UT, in whom we previously reported higher insulin sensitivity and reduced lipid-induced insulin resistance in T compared with UT [4]. Figure 1a depicts a representative trace of leak respiration rates upon increasing amounts of oleate in the permeabilised muscle fibres from T and UT. Interestingly, skeletal muscle fibres from T were more sensitive to FA-induced uncoupling as exemplified by a lower EC50—the concentration of FA at which 50% of maximal uncoupling occurs—compared with muscle fibres from UT (*p* = 0.011 for T vs UT; Fig. 1b). Interestingly, skeletal muscle insulin sensitivity, defined here as the rate of insulin-stimulated whole-body glucose disposal, was inversely correlated with the sensitivity to FA-induced uncoupling in T and UT (*r* = −0.502, *p* = 0.034; Fig. 1c), indicating that an increased sensitivity for FA-induced uncoupling (lower EC50) is related to increased insulin sensitivity. We have previously shown that these same T are partially protected against lipid-induced insulin resistance. Thus, insulin-stimulated non-oxidative glucose disposal (NOGD) is preserved upon lipid infusion in T compared with UT [4]. Interestingly, the EC50 for FA-induced uncoupling negatively correlated with NOGD upon lipid infusion (*r* = −0.624, *p* = 0.013; Fig. 1d), suggesting that a higher sensitivity to FA-induced uncoupling in T contributes to the ‘protection’ of these individuals against a lipid-induced reduction of insulin sensitivity [4]. We did not, however, find a difference in maximal FA-induced uncoupling (*V*_max_ from the fitted FA concentration–response curves) between the T and UT (*p* = 0.422 for T vs UT; Fig. 1e).

We then investigated what could underlie the enhanced sensitivity for FA-induced uncoupling (lower EC50) observed in T at baseline compared with UT. ANT1 has been implicated in mitochondrial leak-dependent respiration and FA-induced uncoupling [13, 31]. Expression of *ANT1* mRNA was significantly elevated in T compared with UT (*p* = 0.013 for T vs UT; Fig. 1f) and protein abundance of ANT1 appeared to be elevated (Fig. 1g, h), although this difference did not reach statistical significance (*p* = 0.10).

### Sensitivity for FA-induced uncoupling is reduced in ZDF rats

Triggered by the relationship between FA-induced uncoupling and acute (lipid-induced) insulin resistance observed in human skeletal muscle, we next chose to focus on the more clinically relevant disease state of insulin resistance and diabetes. For this purpose, we turned to a well-established genetic animal model of insulin resistance and diabetes, ZDF (*fa*/*fa*) rats. To fully exclude the influence of mitochondrial density on the assessment of FA-induced uncoupling, we decided to study FA-induced uncoupling in equal amounts of isolated mitochondria from ZDF vs wild-type control rats. As anticipated, ZDF rats had significantly higher body mass and fasting blood glucose levels compared with wild-type control rats (data not shown).

**Fig. 2:**
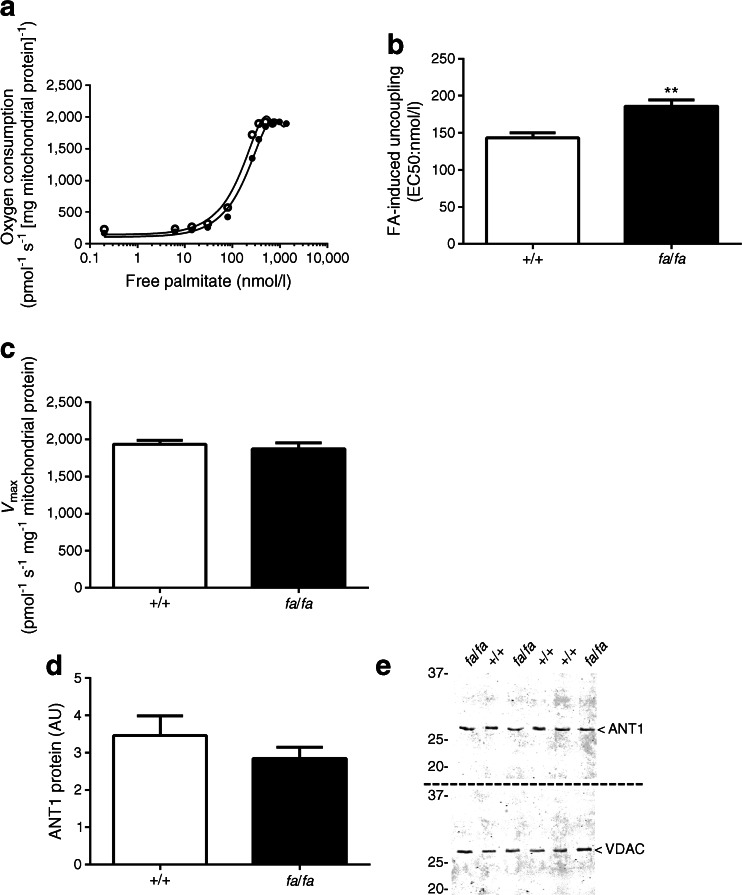
(**a**) Typical experiment assessing FA-induced (palmitate) uncoupling in isolated skeletal muscle mitochondria from wild-type control (*+*/*+*) and ZDF (*fa*/*fa*) rats. (**b**) Sensitivity to FA-induced uncoupling (EC50) in isolated skeletal muscle mitochondria from ZDF rats compared with wild-type control rats (*n* = 4 per group). (**c**, **d**) Maximal palmitate-induced uncoupling (*V*_max_) (**c**) and ANT1 protein levels (shown in arbitrary units [AU]) (**d**) in isolated skeletal muscle mitochondria from wild-type controls and ZDF rats (*n* = 8 per group). (**e**) Representative blots for protein abundance. Two separate gels and blots were used for ANT1 and VDAC as indicated by the dashed line. VDAC was used to quantify equal protein loading during the western blot analysis. White circles and bars, wild-type control rats; black circles and bars, ZDF rats. Free palmitate concentrations were plotted on a logarithmic scale for the representative trace of EC50 in (**a**). Data are presented as mean ± SEM. **p < 0.01, T vs UT

Figure 2a depicts a representative trace of the leak respiration rate at increasing amounts of free palmitate in mitochondria isolated from the gastrocnemius of wild-type control and ZDF rats. Mitochondria from ZDF rats displayed decreased sensitivity for FA-induced uncoupling, as demonstrated by a right shift in the titration curve compared with control mitochondria. This right shift resulted in a 26% increased EC50 in the ZDF rats (*p* = 0.008; Fig. 2b), suggesting an inverse relationship between sensitivity to FA-induced uncoupling and insulin resistance and diabetes in skeletal muscle mitochondria. No difference was observed between the genotypes in *V*_max_ (*p* = 0.528; Fig. 2c). We next investigated whether these observed effects could be attributed to altered levels of ANT1 protein. Contrary to our hypothesis, ANT1 protein abundance was not significantly reduced in skeletal muscle mitochondria of ZDF rats compared with the wild-type control rats (*p* = 0.30; Fig. 2d, e).

**Fig. 3:**
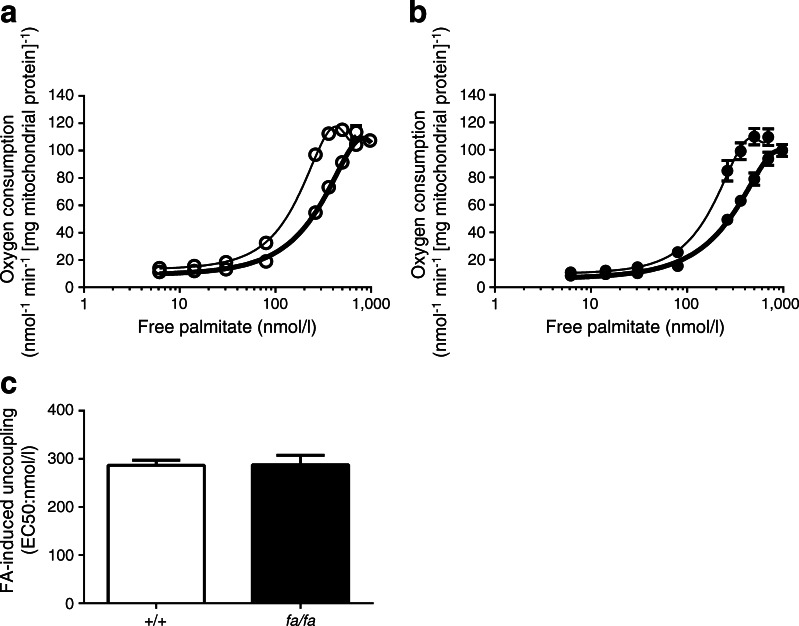
(**a**, **b**) Sensitivity to FA-induced uncoupling (see Fig. 2a) in the absence (thin line) or presence (thick line) of the ANT inhibitor CATR, in wild-type control (*+*/*+*) rats (white circles) (**a**) and ZDF (*fa*/*fa*) rats (black circles) (**b**) was determined. (**c**) EC50 values in the wild-type control (white bar) and ZDF rats (black bar) in the presence of CATR. Free palmitate concentrations were plotted on a logarithmic scale in (**a**) and (**b**) (*n* = 4 per group). Data are presented as mean ± SEM

Since ANT1 protein levels could not explain the decreased sensitivity for FA-induced uncoupling in ZDF rats, we next examined the contribution of ANT1 activity to FA-induced uncoupling directly by chemical inhibition of ANT1 (using CATR) in mitochondria isolated from the gastrocnemius. Inhibition of ANT1 activity significantly decreased the sensitivity for FA-induced uncoupling in ZDF and wild-type control rats (*p* = 0.0001 and *p* = 0.014; *+*/*+* vs *fa*/*fa* rats; Fig. 3a, b). Interestingly, however, the difference in EC50 between the wild-type control and ZDF rats (see Fig. 2b) disappeared upon incubation with the ANT inhibitor (*p* = 0.971; Fig. 3c). These data strongly demonstrate that inhibition of ANT1 activity, in general, decreases the sensitivity for the uncoupling effects of FAs (irrespective of metabolic status and genotype). We found no difference in oxidative phosphorylation capacity of the isolated mitochondria when comparing the genotypes (Table 1).

**Table 1:** Oxygen consumption in isolated skeletal muscle mitochondria from ZDF and wild-type control rats and C2C12 myotubes

Respiration state	Rat isolated skeletal muscle mitochondria			C2C12 myotubes		
	Wild-type rats (*+*/*+*)	ZDF rats (*fa*/*fa*)	*p* value	si-NC (C2C12)	si-*Ant1* (C2C12)	*p* value
State 3^a^	228.1 ± 22.2	225.6 ± 21.5	0.94	60.3 ± 8.4	58.2 ± 6.5	0.85
State 4o^b^	11.7 ± 1.3	11.6 ± 0.8	0.96	13.2 ± 0.6	11.2 ± 0.6	0.08
State U^c^	362.8 ± 34.6	383.6 ± 31.7	0.70	118.7 ± 8.6	111.1 ± 4.8	0.48

Values are means ± SEM

Oxygen consumption ([nmol O_2_] min^−1^ [mg protein]^−1^) was measured in isolated skeletal muscle mitochondria from wild-type control (*+*/*+*) rats (*n* = 4) and ZDF (*fa*/*fa*) rats (*n* = 4) and day 7 C2C12 myotubes without (negative control [si-NC], *n* = 3) and with (si-*Ant1*, *n* = 3) siRNA-mediated knockdown of ANT1

^a^State 3 reflects maximal ADP-stimulated respiration

^b^State 4o represents oligomycin-insensitive oxygen consumption

^c^State U depicts the maximal uncoupled respiration after titration of the protonophore carbonyl cyanide 4-(trifluoromethoxy)phenylhydrazone (FCCP)

### siRNA-mediated silencing of *Ant1* decreased sensitivity to FA-induced uncoupling and insulin in C2C12 myotubes

**Fig. 4:**
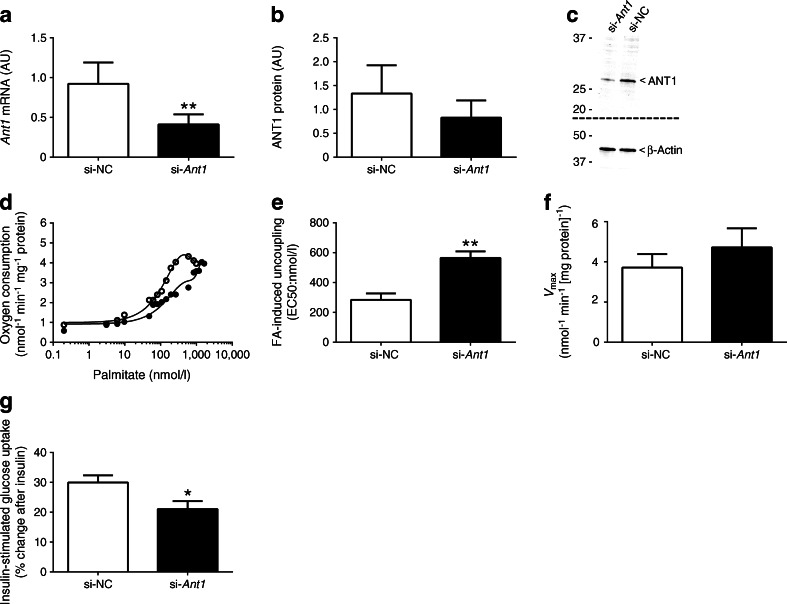
(**a**, **b**) Abundance of *Ant1* mRNA (**a**) and levels of ANT1 protein (**b**) after transfection with negative control siRNA (si-NC) or *Ant1* siRNA (si-*Ant1*) in differentiated C2C12 myotubes (*n* = 4 per group) (shown in arbitrary units [AU]). (**c**) Representative blots for protein abundance. Two separate gels and blots were used for ANT1 and β-actin as indicated by the dashed line. (**d**) Typical experiment assessing FA-induced (palmitate) uncoupling in differentiated, permeabilised C2C12 myotubes after transfection with either si-NC or si-*Ant1*. (**e**) Sensitivity to FA-induced uncoupling (EC50) in si-NC- and si-*Ant1*-transfected C2C12 myotubes (*n* = 3 per group). (**f**) Maximal palmitate-induced uncoupling (*V*_max_) in si-NC- and si-*Ant1*-transfected C2C12 myotubes (*n* = 3 per group). (**g**) Percentage change in insulin-stimulated glucose uptake in si-NC- and si-*Ant1*-transfected C2C12 myotubes (*n* = 9 per group). Palmitate concentrations were plotted on a logarithmic scale for the representative trace of EC50 in (**d**). mRNA expression data were normalised to the reference gene *Rn18s*. α-Actin was used to verify equal protein loading during the western blot analyses. White circles and bars, si-NC; black circles and bars, si-*Ant1*. Data are presented as mean ± SEM. *p<0.05 and **p<0.01 for si-*Ant1* vs si-NC

To further investigate the putative role of ANT1 in FA-induced uncoupling, we aimed to reduce ANT1 expression in differentiated C2C12 myotubes. To avoid interference with overall ATP synthesis, we achieved a partial knockdown of ~60% at the mRNA level (*p* = 0.002; Fig. 4a) and ~40% reduction at the protein level (*p* = 0.052; Fig. 4b, c). *Ant1* gene silencing in differentiated C2C12 myotubes had no aberrant effects on oxidative phosphorylation capacity (Table 1), indicating that maximal ATP synthesis rates were unaffected. Figure 4d depicts a representative trace of the leak respiration rate upon increasing amounts of palmitate in fully differentiated C2C12 myotubes transfected with negative control (si-NC) or *Ant1* siRNA (si-*Ant1*). The reductions in *Ant1* mRNA and ANT1 protein abundance sufficed to reduce the sensitivity to FA-induced uncoupling in C2C12 myotubes as demonstrated by a right shift in the titration curves (Fig. 4d) and a 48.9% increase in the EC50 (*p* = 0.008; Fig. 4e). We observed no differences in *V*_max_ between si-NC and si-*Ant1* (*p* = 0.410; Fig. 4f). Finally, we examined whether reduced ANT1 protein abundance also brought about reduced skeletal muscle insulin sensitivity in C2C12 myotubes. While the insulin response remained intact in both conditions (si-NC: 2.36 ± 0.11 to 3.36 ± 0.09 disintegrations per minute [DPM]/mg protein, *p* < 0.001, basal vs insulin-stimulated; si-*Ant1*: 2.50 ± 0.12 to 3.19 ± 0.15 DPM/mg protein, *p* < 0.01, basal vs insulin-stimulated), the partial knockdown of ANT1 significantly blunted this insulin response compared with control (*p* = 0.025; Fig. 4g).

## Discussion

Endurance exercise training and insulin sensitivity are inextricably linked. We have recently demonstrated that high mitochondrial oxidative capacity (due to chronic endurance exercise training)—both reflected as elevated mitochondrial density and intrinsic function—partially prevented lipid-induced insulin resistance [4]. The underlying mechanisms for this phenomenon are not fully understood. In addition to our findings, in vivo data has demonstrated that endurance-trained muscle exhibits increased substrate oxidation, as well as increased mitochondrial uncoupling [5], resulting in reduced mitochondrial efficiency and increased capacity to waste excess energy. In recent years, the renaissance of research in brown adipose tissue has put a spotlight on this tissue as a thermogenic weapon in the war on obesity and diabetes. However, reducing fat in humans via thermogenesis may require more than brown adipose tissue, an organ that has primarily evolved to maintain core body temperature [32]. Exciting new evidence has revived interest around the thermogenic potential of skeletal muscle [33] by opening the door on the possibility that muscle significantly contributes to non-shivering thermogenesis as a means to maintain substrate metabolism and glucose homeostasis in settings of excess energy (e.g. FAs) with a low demand for ATP production.

In the present study, we demonstrated that elevated sensitivity to FA-induced uncoupling (lower EC50) at baseline was associated with higher insulin sensitivity in endurance-trained compared with sedentary young men. This increased sensitivity to FA-induced uncoupling was further associated with retention of insulin-stimulated glycogen storage (higher non-oxidative glucose disposal [NOGD]) after a lipid infusion. In other words, our data suggest that enhanced sensitivity to FA-induced uncoupling (in the context of chronic endurance exercise training) partially protects skeletal muscle from lipid-induced development of insulin resistance.

In line with our findings, another study demonstrated that a short-term lipid infusion significantly reduced inner mitochondrial membrane potential in healthy (normal glucose tolerance) individuals [34]; this was presumably linked to an increase in uncoupling by the excess FAs. Also in agreement with our data was the fact that mitochondrial content and morphology did not change after lipid infusion and there were no significant changes in citrate synthase activity or total ATP content [34]. In parallel with the positive association between insulin sensitivity and FA-induced uncoupling observed in our human cohort, isolated muscle mitochondria from insulin-resistant ZDF rats displayed a right shift in the FA titration curve, indicating a decreased sensitivity to FA-induced uncoupling.

Although mitochondrial ANT1 protein levels were not statistically different in trained vs sedentary humans and ZDF vs wild-type control rats, the difference in sensitivity to FA-induced uncoupling between mitochondria from ZDF and wild-type controls was abolished upon chemical inhibition of ANT1 activity. This finding suggests that the difference between genotypes originates from FA-induced uncoupling due to ANT1 activity. In this context, almost a decade ago it was demonstrated that ~50% of mitochondrial proton leak can be attributed to ANT1 in murine muscle and that ANT1 can be activated by FAs [31]. Recent work has also demonstrated that acetylation of ANT1 could have dramatic physiological effects on ANT activity and that dysregulation of acetylation of mitochondrial proteins such as ANT1 could therefore be related to changes in mitochondrial function that are associated with insulin resistance [35].

While these in vivo experiments are useful for investigating whole-body physiology, an in vitro loss-of-function model serves to isolate downstream effects of reduction of a singular gene. Thus, we next reduced *Ant1* expression in C2C12 myotubes and studied the sensitivity to FA-induced uncoupling, as well as insulin-stimulated glucose uptake. Since ANT1 plays a crucial role in ADP/ATP exchange across the inner mitochondrial membrane, complete ablation of *Ant1* would lead to serious alterations in oxidative phosphorylation and ATP synthesis rates. Indeed, mice lacking ANT1 are characterised by cardiomyopathy and mitochondrial myopathy of limb muscles [36]. Therefore, we specifically aimed for a partial knockdown of ANT1. By adapting the transfection protocol, we ultimately achieved a 38% reduction at the protein level, which proved not to be rate limiting for maximal ADP-stimulated respiration.

As anticipated, congruent with our findings using the chemical ANT inhibitor CATR in isolated mitochondria to decrease ANT activity, partial knockdown of ANT1 in C2C12 myotubes shifted the titration curve for FA-induced uncoupling to the right (increased EC50), thereby demonstrating reduced sensitivity. The reduction in ANT1 also resulted in a diminished insulin-stimulated glucose uptake compared with controls.

The mechanistic link between FA-induced uncoupling and insulin sensitivity may be related to the production of reactive oxygen species, which was previously identified as the common denominator and causal factor in several cellular models of insulin resistance [37] and which is also associated with insulin resistance in humans [38, 39]. Hence, at the expense of a slight inefficiency in ATP production, mild mitochondrial (FA-induced) uncoupling may be a tool to control the formation of reactive oxygen species when facing a high supply of fatty acids, thereby preserving insulin sensitivity. It should be noted that opening of the mitochondrial permeability transition pore (MPTP) has also been linked to insulin resistance in skeletal muscle [40]. Since ANT is an important regulatory component of the MPTP, knockdown of ANT1 in C2C12 muscle cells may also promote MPTP opening thereby decreasing insulin sensitivity. However, since the partial knockdown of ANT1 that we achieved in the C2C12 muscle cells did not affect oxidative phosphorylation and ATP synthesis in the current study, opening of the MPTP is less likely.

Taken together, these data demonstrate the importance of ANT1 activity in maintaining insulin responsiveness via its role in FA-induced mitochondrial uncoupling. Endurance-trained athletes have superior insulin sensitivity (vs matched sedentary controls) in a setting of elevated muscle lipid content, high basal TCA cycle flux and low basal mitochondrial efficiency (elevated uncoupling) [5]. These findings highlight the potential of FA-induced uncoupling via ANT1 as a target for improving myocellular insulin sensitivity in settings of energy excess.

## Abbreviations

ANT1: Adenine nucleotide translocase 1
CATR: Carboxyatractyloside
FA: Fatty acid
MPTP: Mitochondrial permeability transition pore
NOGD: Non-oxidative glucose disposal
T: Endurance-trained male athletes
TCA: Tricarboxylic acid
UCP: Uncoupling protein
UT: Untrained or sedentary young men
VDAC: Voltage-dependent anion channel
ZDF: Zucker diabetic fatty
